# Genes regulating levels of ω‐3 long‐chain polyunsaturated fatty acids are associated with alcohol use disorder and consumption, and broader externalizing behavior in humans

**DOI:** 10.1111/acer.14916

**Published:** 2022-08-07

**Authors:** Fazil Aliev, Peter B. Barr, Andrew G. Davies, Danielle M. Dick, Jill C. Bettinger

**Affiliations:** ^1^ Department of Psychology Virginia Commonwealth University Richmond Virginia USA; ^2^ Department of Psychiatry & Behavioral Sciences SUNY Downstate Health Sciences University Brooklyn New York USA; ^3^ Department of Pharmacology and Toxicology Virginia Commonwealth University Richmond Virginia USA; ^4^ Virginia Commonwealth University Alcohol Research Center Richmond Virginia USA

**Keywords:** alcohol use disorder, externalizing, LC‐PUFA, omega‐3 polyunsaturated fatty acid

## Abstract

**Background:**

Individual variation in the physiological response to alcohol is predictive of an individual's likelihood to develop alcohol use disorder (AUD). Evidence from diverse model organisms indicates that the levels of long‐chain polyunsaturated omega‐3 fatty acids (ω‐3 LC‐PUFAs) can modulate the behavioral response to ethanol and therefore may impact the propensity to develop AUD. While most ω‐3 LC‐PUFAs come from diet, humans can produce these fatty acids from shorter chain precursors through a series of enzymatic steps. Natural variation in the genes encoding these enzymes has been shown to affect ω‐3 LC‐PUFA levels. We hypothesized that variation in these genes could contribute to the susceptibility to develop AUD.

**Methods:**

We identified nine genes (*FADS1*, *FADS2*, *FADS3*, *ELOVL2*, *GCKR*, *ELOVL1*, *ACOX1*, *APOE*, and *PPARA*) that are required to generate ω‐3 LC‐PUFAs and/or have been shown or predicted to affect ω‐3 LC‐PUFA levels. Using both set‐based and gene‐based analyses we examined their association with AUD and two AUD‐related phenotypes, alcohol consumption, and an externalizing phenotype.

**Results:**

We found that the set of nine genes is associated with all three phenotypes. When examined individually, *GCKR*, *FADS2*, and *ACOX1* showed significant association signals with alcohol consumption. *GCKR* was significantly associated with AUD. *ELOVL1* and *APOE* were associated with externalizing.

**Conclusions:**

Taken together with observations that dietary ω‐3 LC‐PUFAs can affect ethanol‐related phenotypes, this work suggests that these fatty acids provide a link between the environmental and genetic influences on the risk of developing AUD.

## Graphical abstract

Levels of omega‐3 long chain polyunsaturated fatty acids affect the physiological response to alcohol. We tested nine genes that regulate levels of these lipids for association with alcohol use disorder, alcohol consumption and externalizing phenotypes. We found that the set of genes was associated with all three phenotypes, and most of the genes in the set were associated with individual phenotypes. These findings suggest that levels of omega‐3 fatty acids may be important in the development of problematic alcohol use.

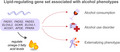

## INTRODUCTION

The misuse of alcohol is a major social and healthcare problem. Alcohol use is linked to significant negative health outcomes; the World Health Organization estimates that alcohol consumption is the cause of 5% of the global disease burden (World Health Organization (Geneva), 2018), and alcohol use contributes to 3 million deaths per year worldwide (World Health Organization (Geneva), 2018). The risk underlying the development of alcohol use disorder (AUD) has both genetic and non‐genetic components; each is responsible for approximately half of the lifetime liability to develop AUD (Prescott & Kendler, 1999). We are interested in the interface of genetics and environment in AUD liability. We recently demonstrated that an environmental factor, dietary long‐chain polyunsaturated omega‐3 fatty acids (ω‐3 LC‐PUFAs), plays an important role in acute behavioral responses to alcohol in invertebrates and mammals (Raabe et al., 2014; Wolstenholme et al., 2018). We found that ω‐3 LC‐PUFAs in the diet interact with the genetic background in mice to modulate voluntary consumption of alcohol (Wolstenholme et al., 2018). In human adolescents, serum ω‐3 LC‐PUFAs levels were associated with alcohol sensitivity (Edwards et al., 2019). Together, these studies support the importance of ω‐3 LC‐PUFAs in alcohol phenotypes.

While diet is the major influencer of ω‐3 PUFA levels in humans, approximately a quarter of the variation in ω‐3 PUFA levels can be explained by genetics (Harris et al., 2012). There are common genetic variants of genes encoding proteins involved in converting ALA to the ω‐3 LC‐PUFAs, and some of these can significantly influence the tissue and serum levels of ω‐3 LC‐PUFAs (Al‐Hilal et al., 2013; Alsaleh et al., 2014; Baylin et al., 2007; Gillingham et al., 2013; Huang et al., 2014; Lemaitre et al., 2011; Mathias et al., 2010; Plourde et al., 2009; Schaeffer et al., 2006; Tanaka et al., 2009). ALA is converted to EPA through actions of the Δ6 desaturase *FADS2*, the elongase *ELOVL1*, and the Δ5 desaturase *FADS1*. EPA is converted into DPA by the elongase *ELOVL2*, and DPA is converted to DHA using the acyl‐CoA oxidase *ACOX1*. Functional allelic variation in these genes would be predicted to influence ω‐3 LC‐PUFA levels, and indeed, a number of studies have identified allelic variation in *FADS1*, *FADS2*, *ELOVL2*, and four additional genes, *FADS3*, *GCKR*, *APOE*, and *PPARA*, as being associated with significant differences in ω‐3 LC‐PUFA levels in humans (Al‐Hilal et al., 2013; Alsaleh et al., 2014; Baylin et al., 2007; Gillingham et al., 2013; Huang et al., 2014; Lemaitre et al., 2011; Mathias et al., 2010; Plourde et al., 2009; Schaeffer et al., 2006; Tai et al., 2005; Tanaka et al., 2009). To date, the contributions of genes regulating ω‐3 LC‐PUFA levels have not been directly tested for association with alcohol phenotypes in humans. We therefore examined these nine genes for association with three key alcohol‐related outcomes available in large samples: alcohol consumption, alcohol use disorder, and externalizing (a composite phenotype consisting of disorders and behaviors characterized by behavioral under control, including alcohol problems). We hypothesized that variation in some or all of these genes affecting ω‐3 LC‐PUFA levels would be associated with one or more of these AUD‐related phenotypes.

## MATERIALS AND METHODS

We analyzed genotypic data for the genes of interest from genome‐wide association data available on our three alcohol‐related phenotypes: alcohol consumption, alcohol use disorder, and externalizing. A full list of the GWAS and their composite samples are presented in Table 1.

**TABLE 1:** Summary of GWAS summary statistics for alcohol‐related phenotypes

Phenotype	Cohort: Original phenotypes	Max *N*	*h* ^2^ (SE)	Mean χ^2^	LDSC intercept	References
Alcohol consumption	GSCAN: drinks per week MVP: AUDIT‐C	738,029	0.048 (0.002)	1.560	0.941	Kranzler et al. (2019), Liu et al. (2019)
Alcohol use disorders	PGC: alcohol dependence MVP: AUD UKB: AUDIT‐P	343,249	0.051 (0.003)	1.396	1.021	Kranzler et al. (2019), Sanchez‐Roige et al. (2019), Walters et al. (2018)
Externalizing	UKB + PGC: problem alcohol use PGC: ADHD ICC: lifetime cannabis use GSCAN: lifetime smoking initiation SSGAC: number of sexual partners SSGAC: age of first sexual intercourse SSGAC: general risk tolerance	1,492,085	0.061 (0.002)	3.114	1.115	Karlsson Linnér et al. (2019)

*Notes:* The statistics reported in this table were all estimated with LD Score regression (LDSC) with heritability (*h* ^*2*^) is on the observed scale. LDSC Intercept is the estimated LD Score regression intercept.

Abbreviations: ADHD, attention‐deficit hyperactivity disorder; AUD, alcohol use disorder; AUDIT‐C, Alcohol Use Disorder Identification Test Consumption Scale; AUDIT‐P, Alcohol Use Disorder Identification Test Problem Scale; GSCAN, GWAS and Sequencing Consortium of Alcohol and Nicotine Use; ICC, International Cannabis Consortium; MVP, Million Veterans Program; PGC, Psychiatric Genomics Consortium; SSGAC, Social Science Genetic Association Consortium; UKB, UK Biobank.

### Alcohol consumption

We generated GWAS by meta‐analyzing two publicly available, large‐scale GWAS of alcohol consumption. The first dataset comes from the GWAS & Sequencing Consortium of Alcohol and Nicotine use (GSCAN) analysis of drinks per week (without the 23&me sub‐sample) in approximately 550 K individuals of European ancestries (Liu et al., 2019). The second GWAS comes from the Million Veterans Program (MVP) GWAS of the first three items related to alcohol consumption in the Alcohol Use Disorder Identification Test (AUDIT), referred to as AUDIT‐C, in approximately 200 K individuals of European ancestries (Kranzler et al., 2019). These sets of summary statistics were highly correlated (*r*_g_ = 0.70), and we used a sample size‐weighted meta‐analysis in METAL (Willer et al., 2010), resulting in the total sample size of ~750 K individuals.

### Alcohol use disorder (AUD)

We created GWAS data for AUD by meta‐analyzing three sets of summary statistics: AUD as assessed using DSM‐IV Alcohol dependence criteria measured in the Million Veterans Program (*N* ~ 200 K) (Kranzler et al., 2019; Zhou et al., 2020), DSM‐IV Alcohol Dependence criteria as analyzed by the Psychiatric Genomics Consortium (*N* ~ 29 K) (Walters et al., 2018), and the problem subscale of the AUDIT analyzed in the UK Biobank (AUDIT‐P, *N* ~ 121 K) (Sanchez‐Roige et al., 2019), again using a sample size‐weighted meta‐analysis because of the relatively strong genetic overlap between each (*r*_g_ = 0.60 to 1.00). UK Biobank AUDIT‐P is based on items 4 to 10 of the AUDIT, which is summed to produce an overall problem scale score. This score was log10 transformed in analyses to approximate a normal distribution. Our final sample size was *N* ~ 350 K individuals of European ancestries.

### Externalizing

Externalizing is an umbrella term used to describe a variety of behaviors/problems related to behavioral disinhibition that are correlated at the phenotypic and genetic levels (Barr & Dick, 2020). We derived our genetic data for externalizing from a recent multivariate, common factor GWAS (Karlsson Linnér et al., 2021). The latent externalizing factor included seven GWAS related to behavioral under control as inputs: ADHD (*N* = 53,293; Demontis et al., 2019), problematic alcohol use (*N* = 164,121; Sanchez‐Roige et al., 2019; Walters et al., 2018), lifetime cannabis use (*N* = 186,875; Pasman et al., 2018), age at first sexual intercourse (*N* = 357,187; Karlsson Linnér et al., 2019), number of sexual partners (*N* = 336,379; Karlsson Linnér et al., 2019), general risk tolerance (426,379; Karlsson Linnér et al., 2019), and lifetime smoking initiation (*N* = 1,251,809; Liu et al., 2019). The estimated sample size for the externalizing factor was approximately 1.5 million individuals.

Our primary analyses were gene‐set analyses that tested for overall association with the set of nine genes (*FADS1*, *FADS2*, *FADS3*, *ELOVL2*, *GCKR*, *ELOVL1*, *ACOX1*, *APOE*, *PPARA*) and the three alcohol‐related outcomes. We created a gene set containing all nine genes and ran gene‐set analysis using the *R* COMBAT package (Wang et al., 2017). COMBAT requires only SNP level *p*‐values and correlations between SNPs from ancestry‐matched samples and performs an extended Simes procedure to combine multiple parallel association test results performed by using Gates, Vegas (five tests and combined test), and simpleM methods. COMBAT then creates an overall association *p*‐value.

Subsequently, we performed gene‐based tests with each of the individual genes. We first performed MAGMA gene‐based analysis and gene‐set analysis on the full GWAS input data through FUMA using the alcohol consumption, AUD, and externalizing GWAS results (Watanabe et al., 2017). FUMA uses input GWAS summary statistics to compute gene‐based *p*‐values (gene analysis). The gene‐based *p*‐value for gene analysis is computed for protein‐coding genes by mapping SNPs to genes if SNPs are located within the genes. GWAS results from each of the samples were loaded to FUMA SNP2GENE software for gene‐based analyses.

For robust comparison of FUMA results, we ran FastBAT which performs a set‐based association analysis for human complex traits using summary‐level data from GWAS and linkage disequilibrium (LD) data from a reference sample with individual‐level genotypes. For the reference panel, we used 1000 Genome data. These data agree with the FUMA analysis and appear in Table S1.

## RESULTS

Table 2 shows the results from all of the COMBAT analyses. The nine‐gene set (*FADS1*, *FADS2*, *FADS3*, *ELOVL2*, *GCKR*, *ELOVL1*, *ACOX1*, *APOE*, *PPARA*) was highly associated with all three phenotypes.

**TABLE 2:** COMBAT analyses for alcohol consumption, AUD, and broad externalizing

	Alcohol consumption	Alcohol use disorder	Broad externalizing
COMBAT	<0.00001	0.01416	0.00017
GATES	<0.00001	0.01646	0.00010
VEGAS.max	0.00003	0.01593	0.00013
VEGAS.p0.1	0.00003	0.00648	0.14858
VEGAS.p0.2	0.00003	0.02295	0.24238
VEGAS.p0.3	0.00003	0.02592	0.25939
VEGAS.p0.4	0.00003	0.03348	0.28488
VEGAS. all	0.00003	0.03969	0.37727
simpleM	<0.00001	0.01467	0.00008

Abbreviation: AUD, alcohol use disorder.

Table 3 presents the results from gene‐based analyses. *p*‐Values presented in the table are Bonferroni corrected for multiple testing for both genes (number of protein‐coding genes in gene‐based analyses) and the number of independent signals of three phenotypes (Alcohol Consumption, AUD, and Externalizing). The number of protein‐coding genes used in the FUMA gene analyses was 18,896, 18,891, and 18,318, respectively, for Alcohol Consumption, AUD, and Externalizing. Because the three phenotypes are correlated, we calculated the effective number of independent tests using the *meff* function in the R (version) *poolr* package which uses the method described by Galwey (2009). We found that there were two independent signals, and used this number in our correction. With corrected final *p*‐values for the number of genes and independent phenotypes in Table 3, we considered analyses with *p*‐values <0.05 as significant and with *p*‐values <0.10 as suggestive. Based on these corrected *p*‐values we found that the *GCKR*, *FADS2*, and *ACOX1* genes showed a significant association with alcohol consumption. *FADS1* and *FADS3* showed suggestive signals of association (*p* < 0.1) with alcohol consumption. *GCKR* was significantly associated with AUD. *ELOVL1* was strongly associated with externalizing. *APOE* also was significantly associated with externalizing (*p* = 0.03).

**TABLE 3:** Gene analyses results for alcohol consumption, AUD, and broad externalizing. Values in bold are significant (p‐values < 0.05) or suggestive (p‐values < 0.10)

Gene	Alcohol consumption			Alcohol use disorder			Broad externalizing		
	NSNPS	ZSTAT	*p*	NSNPS	ZSTAT	*p*	NSNPS	ZSTAT	*p*
FADS1	36	2.36	**0.0546**	37	−0.41	1.0000	30	1.40	0.1628
FADS2	160	2.21	**0.0270**	165	−1.21	1.0000	137	0.93	0.3504
FADS3	35	1.79	**0.0736**	32	0.00	0.9966	24	−0.60	1.0000
ELOVL2	163	−0.08	1.0000	120	0.22	0.8248	90	−0.92	1.0000
GCKR	41	9.24	**2.4E‐20**	41	7.00	**2.6E‐12**	34	1.16	0.2470
ELOVL1	4	−2.00	1.0000	7	0.24	0.8088	2	4.49	**7.2E‐06**
ACOX1	139	2.52	**0.0118**	138	0.13	0.8928	96	0.57	0.5718
APOE	6	1.04	0.3000	6	0.62	0.5350	5	2.13	**0.0332**
PPARA	223	0.02	0.9856	196	−0.02	1.0000	112	0.32	0.7512

Abbreviations: NSNPS, the number of SNPs from the corresponding GWAS annotated to that gene that was found in the data and was not excluded based on internal SNP QC; *p*, the gene *p*‐value, using asymptotic sampling distribution; ZSTAT, the *Z*‐value for the gene, based on its (permutation) *p*‐value.

## DISCUSSION

Several lines of evidence suggest that levels of ω‐3 LC‐PUFAs influence acute physiological responses to alcohol (Raabe et al., 2014; Wolstenholme et al., 2018). In mammals, including humans, the majority of ω‐3 long‐chain polyunsaturated fatty acids (ω‐3 LC‐PUFAs) are derived directly from the diet, and levels of dietary ω‐3 LC‐PUFAs strongly influence tissue ω‐3 LC‐PUFA levels (Superko et al., 2013). The major ω‐3 LC‐PUFAs in humans, eicosapentaenoic acid (EPA), docosahexaenoic acid (DHA), and docosapentaenoic acid (DPA), are derived primarily from seafood (Chung et al., 2008; Sun et al., 2007). Humans can also make ω‐3 PUFAs; the short chain ω‐3 PUFA, α‐linolenic acid (ALA), derived from plant sources, can be converted to EPA through a series of elongation and desaturation steps. EPA can be converted to DPA and then to DHA. The conversion of ALA to EPA is inefficient relative to acquiring EPA and DHA from dietary sources, and this conversion is thought to be important in regulating ω‐3 PUFA levels in people who do not eat seafood (Burdge & Wootton, 2003; Goyens et al., 2005, 2006).

Here we tested for association between genes involved in the generation of ω‐3 LC‐PUFAs and genes with allelic variants that modify ω‐3 LC‐PUFA levels, and alcohol‐related phenotypes in humans. We identified nine genes that are directly required for the generation of ω‐3 LC‐PUFAs (*FADS1*, *FADS2*, *ELOVL1*, *ELOVL2*, and *ACOX1*) or for which we found evidence from human studies that they are involved in the regulation of ω‐3 LC‐PUFAs levels (*FADS1*, Al‐Hilal et al., 2013; Gillingham et al., 2013; Lemaitre et al., 2011; Mathias et al., 2010; Schaeffer et al., 2006; Tanaka et al., 2009; *FADS2*, Al‐Hilal et al., 2013; Baylin et al., 2007; Gillingham et al., 2013; Lemaitre et al., 2011; Mathias et al., 2010; Schaeffer et al., 2006; Tanaka et al., 2009; *FADS3*, Huang et al., 2014; Tanaka et al., 2009; *ELOVL2*, Alsaleh et al., 2014; Lemaitre et al., 2011; Tanaka et al., 2009; *GCKR*, Lemaitre et al., 2011; *APOE*, Plourde et al., 2009; and *PPARA*, Tai et al., 2005).

When tested as a set, we found that the nine genes were significantly associated with all alcohol‐related phenotypes examined: alcohol consumption, AUD, and externalizing. We also found that several of these genes were individually significantly associated with different alcohol‐related phenotypes. We found that variation in *GCKR*, *FADS2*, and *ACOX1* was significantly associated with the consumption phenotype, *GCKR* was associated with AUD, and *ELOVL1* and *APOE* were associated with externalizing.

We have previously shown that dietary levels of ω‐3 LC‐PUFAs significantly alter measures of levels of response to alcohol in model organisms; in humans, the level of response to alcohol (LR) is predictive of liability to develop an alcohol use disorder, so we are interested in understanding the factors that modulate LR. We previously found that the ω‐3 LC‐PUFA eicosapentaenoic acid (EPA) is required for the development of acute functional tolerance to EtOH, one component of LR, in the invertebrate model *C. elegans*, and that increasing the levels of EPA enhanced acute functional tolerance (Raabe et al., 2014). We extended these observations to mice and found that dietary supplementation of the ω‐3 LC‐PUFAs EPA and DHA in the form of fish oil also modulated both the acute stimulatory and sedative effects of EtOH, as tested by locomotor activation and loss of righting reflex assays (Wolstenholme et al., 2018). These dietary ω‐3 LC‐PUFAs also affected voluntary EtOH intake in mice in a genotype‐specific manner; in DBA/2J mice, fish oil supplementation changed acute EtOH sedation, but not in C57BL/6J mice, whereas fish oil supplementation changed voluntary EtOH consumption only in C57BL/6J mice (Wolstenholme et al., 2018). We found that in humans, there was a relationship between ω‐3 LC‐PUFA levels and acute level of response to EtOH in the Avon Longitudinal Study of Parents and Children (ALSPAC) dataset: there was an association between ω‐3 LC‐PUFA levels in blood and the initial level of alcohol response as assessed by the Self‐Rating of Effects of Alcohol (SRE) in adolescents. Adolescents with higher ω‐3 LC‐PUFA levels reported a lower acute response to alcohol (Edwards et al., 2019).

In addition to our own observations, several other lines of evidence have converged to strongly implicate dietary ω‐3 LC‐PUFAs in relevant behavioral responses to alcohol. Nutritional supplementation, including EPA and DHA, was associated with improvements in externalizing behavior in a high‐risk group of children (Raine et al., 2016). A positive relationship has been described between dietary intake of ω‐3 LC‐PUFAs and impulse control in adolescents (Darcey et al., 2018). Impulse control is a central component of the broad externalizing phenotype that is associated with problematic alcohol use (Dick et al., 2010), suggesting that ω‐3 LC‐PUFAs may impact both acute effects of EtOH and behavioral mechanisms that are associated with alcohol abuse. ω‐3 LC‐PUFA levels have also been associated with the relapse rate in individuals with substance use disorders; lower levels of plasma ω‐3 LC‐PUFAs correlated with a higher risk of relapse (Buydens‐Branchey et al., 2009). In individuals with substance use disorders who are abstinent, supplementation of dietary ω‐3 LC‐PUFAs was associated with a decrease in cortisol levels and measures of anxiety (Barbadoro et al., 2013). In non‐dependent individuals, ω‐3 LC‐PUFA levels were also correlated with alcohol consumption (Di Giuseppe et al., 2009).

How might levels of ω‐3 LC‐PUFAs affect alcohol responses? ω‐3 LC‐PUFAs have important and diverse functions in the nervous system, and their levels have been reported to be lower in individuals with certain psychiatric illnesses including major depression, which is often co‐morbid with alcohol use problems (reviewed in Freeman et al., 2006; McNamara et al., 2007). LC‐PUFAs are structural components of biological membranes, and the levels of ω‐3 LC‐PUFAs in cell membranes have important consequences on the functions of neurons (Gawrisch & Soubias, 2008; Shaikh et al., 2015). The levels of ω‐3 LC‐PUFAs have significant effects on membrane microarchitecture, which in turn can regulate the activity of membrane‐bound proteins (Fan et al., 2003). Membrane microarchitecture can also change how and if EtOH interacts with its direct targets. For example, the ability of EtOH to interact with its direct target, the SLO‐1 BK potassium channel, is significantly altered by the composition of the membrane in which the channel resides (Yuan et al., 2008). The GABA_A_ receptor is another major direct target of EtOH's actions, and membrane microarchitecture can affect drug binding to GABA_A_ receptors (Nothdurfter et al., 2013). Taken together, these data suggest a model in which ω‐3 LC‐PUFAs levels may modulate both neuronal functions and directly influence the effects of EtOH on the brain.

LC‐PUFAs are also the backbones of several types of lipid signaling molecules, including the eicosanoids, which are responsible for both proinflammatory and anti‐inflammatory actions. The ω‐3 LC‐PUFAs EPA and DHA are the bases of the resolvins, a group of eicosanoids that are involved in the resolution of inflammation, including in the brain (Labrousse et al., 2018; Madore et al., 2020), and decreases in resolvin biosynthesis have been observed in several brain diseases (recently comprehensively reviewed in Dyall et al., 2022). Pro‐inflammatory eicosanoids are generated from the ω‐6 LC‐PUFAs, so it is thought that the ratio of ω‐3/ ω‐6 LC‐PUFAs is important in determining the levels of inflammation. Heavy alcohol drinking causes neuroinflammation (Crews et al., 2013; King et al., 2020; Pascual et al., 2014), suggesting that ω‐3 LC‐PUFA mediated effects on inflammation may therefore be important in modulating these physiological effects of EtOH.

Human genetic studies have also provided clues for the possible roles of ω‐3 LC‐PUFAs in alcohol phenotypes. One of the best‐supported candidate loci for association with human AUD phenotypes is the gene *KLB* (Jorgenson et al., 2017; Sanchez‐Roige et al., 2019; Schumann et al., 2016). Animal studies implicate ω‐3 LC‐PUFAs in the function of *KLB*: Fish oil supplementation significantly increased *KLB* expression (both mRNA and protein) in the liver in mice (Yang et al., 2017). *KLB* is required for the function of *FGF21*, which functions in the brain to regulate alcohol preference in mice (Schumann et al., 2016), and acts as an important lipid metabolism regulator. Fish oil supplementation also increased the signaling of *FGF21* in mice (Yang et al., 2017). In humans, an explicit relationship between dietary EPA and *FGF21* levels was recently described; EPA supplementation increased circulating *FGF21* levels (Escote et al., 2018).

Among the genes that we tested, *GCKR* has previously been shown to be associated with alcohol consumption in the UK Biobank (Clarke et al., 2017), with drinks per week in GSCAN (Liu et al., 2019), and with total AUDIT score in a composite sample of UK Biobank and 23andMe (Sanchez‐Roige et al., 2019). Intriguingly, allelic variation in *GCKR* had been previously been found to be associated in a GWAS from the CHARGE consortium with variation in levels of the ω‐3 LC‐PUFA DPA, an intermediate product of the conversion of EPA to DHA (Lemaitre et al., 2011). However, it is important to note that these previous analyses were based on samples that were also incorporated into the larger meta‐analytic results examined in the current analysis and thus should not be interpreted as independent evidence.

Our findings should be interpreted in the context of the following limitations. Because genetic analyses require large sample sizes for adequate power and the reliable and replicable detection of effects (Hong & Park, 2012), we limited our analyses to large extant samples with genotypic data. This necessarily limits the depth of phenotyping available. Follow‐up analyses should further characterize the pathways by which genetic and environmental factors jointly contribute to ω‐3 LC‐PUFA levels to influence alcohol consumption and related behavioral regulation outcomes in humans. It is also likely that there are additional genes involved in the regulation of ω‐3 LC‐PUFA levels that were not examined here. We confined our analyses to genes for which there is extremely strong evidence of effects on ω‐3 LC‐PUFA levels. We tested genes encoding enzymes in the biosynthetic pathways known to directly generate the three main ω‐3 LC‐PUFAs or that have been shown to affect levels of ω‐3 LC‐PUFAs in humans, but it is very likely that other genes that are not represented in these two groups also regulate ω‐3 LC‐PUFA levels. We predict that such genes may also affect alcohol‐related phenotypes.

Ultimately, our hope in characterizing the pathways that underlie risk for problematic use of alcohol is to assist in the rational development of new preventative measures, interventions, and treatment options. Our data strongly suggest that the genetic regulation of ω‐3 LC‐PUFA levels is important in alcohol‐related phenotypes in humans. There is ample evidence that dietary ω‐3 LC‐PUFAs can also influence alcohol‐related phenotypes in model organisms and in humans (Barbadoro et al., 2013; Buydens‐Branchey et al., 2009; Clarke et al., 2017; Darcey et al., 2018; Di Giuseppe et al., 2009; Edwards et al., 2019; Liu et al., 2019; Raabe et al., 2014; Raine et al., 2016; Sanchez‐Roige et al., 2019; Schumann et al., 2016; Wolstenholme et al., 2018; Yang et al., 2017). Taken together, this body of work strongly supports a more thorough investigation of the physiological roles of both exogenous and endogenous ω‐3 LC‐PUFAs in the development of alcohol use disorder and the related externalizing phenotypes.

## AUTHOR CONTRIBUTIONS

FA and PBB performed the analysis and interpreted findings. AGD, DMD, and JCB were responsible for the study concept, design, and interpretation of findings. All authors contributed to the drafting of the manuscript, and all authors critically reviewed the content and approved the final version for publication.

## FUNDING INFORMATION

The Externalizing Consortium has been supported by the National Institute on Alcohol Abuse and Alcoholism (R01 AA015416—administrative supplement) and the National Institute on Drug Abuse (R01 DA050721). The VCU Alcohol Research Center is supported by the National Institute on Alcohol Abuse and Alcoholism (P50 AA022537).

## CONFLICT OF INTEREST

The authors have no conflicts of interest to declare.

## Supporting information


Table S1
