# Supplementary material for: Genes regulating levels of ω‐3 long‐chain polyunsaturated fatty acids are associated with alcohol use disorder and consumption, and broader externalizing behavior in humans
Source: Alcohol Clin Exp Res. 2022 Aug 7;46(9):1657–64. doi: 10.1111/acer.14916 (PMC9509483; doi:10.1111/acer.14916)
Supplement: Supplementary file 1 — Table S1 [file ACER-46-1657-s001.docx]

**Table S1. GCTA FastBAT gene analyses results for Alcohol Consumption, AUD and Broad Externalizing. Values in bold are significant (p-values 〈0.05) or suggestive (p-values 〈0.10)**

|  | **Alcohol Consumption** | | | **Alcohol Use Disorder** | | | **Broad Externalizing** | | |
| --- | --- | --- | --- | --- | --- | --- | --- | --- | --- |
| **GENE** | **NSNPS** | **Chisq(Obs)** | **P** | **NSNPS** | **Chisq(Obs)** | **P** | **NSNPS** | **Chisq(Obs)** | **P** |
| **FADS1** | 74 | 164.23 | **0.0424** | 73 | 43.95 | 1 | 53 | 70.3 | 0.4046 |
| **FADS2** | 92 | 190.44 | **0.052** | 90 | 68.97 | 1 | 62 | 74.44 | 0.5062 |
| **FADS3** | 81 | 162.98 | **0.0614** | 79 | 56.24 | 1 | 55 | 54.55 | 0.7738 |
| **ELOVL2** | 42 | 41.63 | 0.8516 | 39 | 35.31 | 1 | 21 | 12.18 | 1 |
| **GCKR** | 35 | 939.7 | **5.72E-27** | 40 | 413.43 | **1.52E-10** | 29 | 75.23 | **0.021** |
| **ELOVL1** | 16 | 19.91 | 0.5 | 16 | 36.71 | 0.0304 | 11 | 75.86 | **3.20E-06** |
| **ACOX1** | 62 | 132.72 | **0.018** | 61 | 86.26 | 0.2586 | 36 | 50.08 | 0.3578 |
| **APOE** | 35 | 42.71 | 0.4964 | 33 | 52.33 | 0.2262 | 23 | 71.69 | **0.0194** |
| **PPARA** | 102 | 147.34 | 0.1788 | 86 | 90.59 | 0.6942 | 35 | 29.47 | 1 |

NSNPS: the number of SNPs from the corresponding GWAS annotated to that gene, Chisq(Obs): observed Chi-squared value, P: the gene p-value. FastBAT used Linkage disequilibrium (LD) pruning based on 1000G reference panel data.
